# Enhancement for well-being is still ethically challenging

**DOI:** 10.3389/fnsys.2014.00072

**Published:** 2014-04-30

**Authors:** Saskia K. Nagel

**Affiliations:** Institute of Cognitive Science, University of OsnabrueckOsnabrueck, Germany

**Keywords:** enhancement, neuro-enhancement, well-being, happiness, ethics, neuro-ethics

*“If we were to ask the question: ‘What is human life's chief concern?’ one of the answers we should receive would be: ‘It is happiness.’ How to gain, how to keep, how to recover happiness is in fact for most men at all times the secret motive of all they do, and of all they are willing to endure.” (William James 1902)*.

Enhancement is generally understood as being intended to improve well-being. The motivation to enhance is the desire to change a person for the better. However, even when increased well-being is the motivation, it is unclear how to morally evaluate any given intervention. Four examples illustrate why any enhancement intervention, including those motivated by the desire to increase well-being, still demands ethical reflection.

## Enhancement—vaguely defined and controversial

Humans have always been fighting, with all the means at their disposal, against disease, pain, and unhappiness, fighting to increase their quality of life. There have been many facts, fictions, and controversies around the enhancement of brain functions in the last 15 years. Ever since the debate started new definitions of enhancement have been proffered, often diverging from each other and leading to debates on a wide field of ethical and social matters (Parens, 1998; Farah et al., 2004; Levy, 2007; Greely et al., 2008; Schermer et al., 2009; Nagel, 2010b). Enhancement interventions come in many varieties: there are manifold methods, goals, motivations, desires, ideals, and values that can invoke heated discussions. Moral deliberation reaches from statements such as those put forward in the President's Council on Bioethics report Beyond Therapy (2003) with an anti-enhancement agenda mainly based on arguments around the concepts of naturalness and dignity, to arguments for the moral obligation to enhance (Harris, 2005; Savulescu, 2005)^1^. This wide variety in moral evaluations partly seems to be based on different understandings of the very term. Although “enhancement” is a notoriously vague term, a general consensus of what is meant is often implicitly assumed.

## Motivation and goal: well-being

Here, I will attempt to further a particular understanding of the concept that shall serve to improve mutual understanding of the different positions. Furthermore, I suggest distinguishing what enhancement *is* and *how it is motivated* from how its usage is ethically evaluated. Julian Savulescu and colleagues distinguish various ways of conceptualizing enhancement and propose a “welfarist definition of human enhancement: Any change in the biology or psychology of a person which increases the chances of leading a good life in the relevant set of circumstances. (….) It singles out well-being as one dimension of value that is constitutive of genuine human enhancement.” (Savulescu et al., 2011, 7). Brian Earp and colleagues contrast this welfarist approach with the “augmentative functionalist approach” to show how diminishment can be enhancement (Earp et al., 2014). John Harris elaborates on how enhancement is about making us better people: “Enhancements will be enhancements properly so-called if they make us better at doing some of the things we want to do, better at experiencing the world through all of our senses, better at assimilating and processing what we experience, better at remembering and understanding things, stronger, more competent, more of everything we want to be (…) In terms of human functioning, an enhancement is by definition an improvement on what went before. If it wasn't good for you, it wouldn't be enhancement.” (Harris, 2007, 2ff). Enhancement is understood as generally being *for the better* of people. If it was not for an improvement for the better of an individual we would not call it enhancement. In fact, it is a tautology to say “enhancement for the better” as enhancement implies that it improves a given state, a performance, a capacity, an appearance, but also an experience, a feeling and, importantly: one's evaluations thereof.

## Even when it is for the better it is not always good: motivation does not equal evaluation

A popular slogan about enhancement is that it makes people “better than well” (Elliott, 2003). Back in 1998, Erik Parens asked: “is better always good?” The problem actually is what “the better” really is in the plethora of possible cases of individuals in their unique socio-cultural contexts. How to evaluate what is better for a person, for his or her surrounding, or for the wider social context is a central matter for the normative discussion about it (Nagel, 2010b; Racine, 2010; Glannon, 2011). Hence, it is useful to inquire about the purpose of the particular enhancement intervention. Enhancements are usually not driven by the motivation to have merely *more* of some capacity, but rather by the desire to change for the better^2^. Restricting the concept of enhancement to the addition of capacities or augmentation of function (Bostrom, 2009) does not do justice to the rich variety of forms which enhancement can take, and underestimates the plethora of motivations of people striving for enhancement. The goal of enhancement is improvement. One can argue whether one understands it as improvement beyond the normal, or improvement beyond the natural (Sabin and Daniels, 1994; Daniels, 2000; Buchanan, 2008, 2011)—both understandings lead to their own complex debates, and the concepts themselves require more scrutiny than this article can offer. Each view comes with its problems, and the consequent moral evaluations differ depending on what people feel most committed to (Parens, 2005). First and foremost, the goal of enhancement is about what people strive for most: “How to gain, how to keep, how to recover happiness” as William James put it in 1902. However, while interventions may indeed be motivated by the desire to increase flourishing, it is far from clear that the interventions indeed yield this increase in well-being. This view does not imply a normative evaluation of the enhancement intervention in a particular case. John Harris states that “enhancements *per se* are not ethically problematic: they are unequivocally good, clearly ethical. Unless the downside can be demonstrated and is significant, enhancement has the moral high ground” (Harris, 2005). While one might agree that enhancement *per se* always aims for something good, i.e., well-being, the evaluation of an enhancement varies between individuals depending on their situation. The moral evaluation of the intervention in specific cases does not depend solely on the desired goal of the intervention.

## Ethically challenging even though well-being is the goal

The following examples should help clarify the general notion of enhancement as meaning “enhancement for the better,” and demonstrate how this still leads to complex moral challenges.

1. **The usage of methylphenidate and amphetamine products in children for enhancement purposes, e.g., to improve cognitive functioning:** Despite the fact that data on side effects and effectiveness is sparse, usage of prescription medication for enhancement is increasing (McCabe et al., 2011; Smith and Farah, 2011; Kaye and Darke, 2012). For pediatric enhancement, one can assume that parents act with the best intentions for their children. They aim to foster their children's flourishing and often hope to do so with some form of enhancement. Thus, while striving for the best for their children and thus aiming at “enhancement for the better,” they might still risk the child's current and future well-being (e.g., Urban and Gao, 2014). Over and above the individual impact, there are many pressing social issues surrounding pediatric neuro-enhancement (Singh and Kelleher, 2010; Graf et al., 2013).
2. **Using neuro-technologies and psychopharmacology to induce plasticity for general-purpose enhancement:** Trans-cranial electrical stimulation like transcranial direct current stimulation and transcranial random noise stimulation can be employed as tools to induce neuroplastic cortical excitability alterations (Nitsche and Paulus, 2011; Cohen Kadosh, 2013; Snowball et al., 2013; for discussions see Cohen Kadosh et al., 2012; Fitz and Reiner, 2013; Davis and van Koningsbruggen, 2014; Krause and Cohen Kadosh, 2014). Furthermore, the commonly used anticonvulsant and mood stabilizer Valproat has recently been shown to induce plasticity, thereby reopening critical periods for learning (Gervain et al., 2013). These technologies seem to promise general enhancement potential by targeting neuronal plasticity. They certainly can be used for the better of the users. Crucially, however, this depends on the risk and side-effect profile. Moreover, ethical questions go beyond purely individual reasoning and must consider normative questions related to the values that a society wants to promote. This requires a cautious approach that can only be hinted at here. Paramount is the realization that “enhancement for the better” still offers challenges that require ethical reasoning.
3. **Using psychopharmacological agents to erase or modify memory:** Unpleasant memories diminish life quality. Especially individuals who have experienced trauma or shock can suffer from horrible, haunting memories. Manipulating such memories or their emotional intensity promises to increase well-being. While critics argue that for individual, social, and legal reasons “routinely interfering with the memories of trauma survivors and witnesses is highly questionable.” (President's Council on Bioethics, chapter 5, IIC, Schacter, 2001), the motivation for even healthy people to seek memory blunting (e.g., with beta-blockers) is the desire to flourish. For the purpose of the present argument, the key aspect is this: Memory blunting as enhancement may help well-being but still requires ethical reasoning (Glannon, 2006; Kolber, 2006).
4. **Enhancing by amputation?:** A particularly challenging case for describing what enhancement could mean if one strongly stretches the concept into the realm of treatment, is an intervention in cases of Body Integrity Identity Disorder (BIID). BIID is a rare disorder in which patients suffer a complete lack of identification with a healthy limb and obsessively desire its amputation (First, 2005). They suffer sometimes so strongly that they will harm themselves to get rid of the unwanted limb. In these cases, amputation reduces anxiety, relieves suffering, and increases the amputees' subjective quality of life. Despite the unclear medical categorization, and despite the fact that these patients suffer from a disorder, and thus interventions qualify as treatment rather than enhancement, the dilemma is strong: There are patients with BIID who can decide autonomously for elective amputation that harms their physical body but enhances their well-being (Bayne and Levy, 2005). Approaching such an amputation as enhancement is provocative, shatters intuitions, and thereby demands clarification of what is meant by enhancement.

## How to proceed?

Various situations of enhancement can lead to more flourishing. However, this does not foreclose ethical discussions. Ethical dilemmas emerge even if the goal of the intervention is increased well-being. I agree with Erik Parens who notes that “some (…) think the term enhancement is so freighted with erroneous assumptions and so ripe for abuse that we ought not even to use it. My sense is that if we didn't use enhancement, we would end up with another term with similar problems” (Parens, 1998, 2). Clearly, there is still work to be done to clarify what we mean with enhancement. Future deliberation should include two issues that have not yet been considered sufficiently in current debates: (1) Studying the nature of well-being, and how it can be increased (Parfit, 1984; Kagan, 1992; Nussbaum and Sen, 1993; Scanlon, 1998), and (2) probing public attitudes on enhancement to allow regulation and political decision-making to be nuanced and sound (Nadler and Reiner, 2010; Fitz et al., 2013). While working on this, it is worthwhile to avoid clamoring for a binary position either “for” or “against” enhancement in general, and instead foster sensitive discussions about the concerns and idiosyncrasies of each different case.

### Conflict of interest statement

The author declares that the research was conducted in the absence of any commercial or financial relationships that could be construed as a potential conflict of interest.

## Footnotes

^1^Peter Reiner provides a lucid analysis of how this discussion is deeply bio-political, and driven by strong intuitions of those at the extremes (Reiner, 2013).

^2^I have argued elsewhere that “more” can even have negative effects on well-being (Nagel, 2010a). This refers not only to side effects and long-term effects but also to the potential burden of increased responsibility. Responding to this, John Danaher has argued that this objection does not justify the project to “forestall or delay the enhancement” project (Danaher, 2013). However, forestalling the enhancement project is not the goal of expressing this concern. The goal is to illustrate the need for closer scrutiny of the diverse influences of enhancement on well-being.
